# Epigenetic outlier profiles in depression: A genome-wide DNA methylation analysis of monozygotic twins

**DOI:** 10.1371/journal.pone.0207754

**Published:** 2018-11-20

**Authors:** Aldo Córdova-Palomera, Helena Palma-Gudiel, Jaume Forés-Martos, Rafael Tabarés-Seisdedos, Lourdes Fañanás

**Affiliations:** 1 Departament de Biologia Evolutiva, Ecologia i Ciències Ambientals, Facultat de Biologia and Institut de Biomedicina (IBUB), Universitat de Barcelona, Barcelona, Spain; 2 Centro de Investigaciones Biomédicas en Red de Salud Mental (CIBERSAM), Instituto de Salud Carlos III, Madrid, Spain; 3 Department of Medicine, University of Valencia and Instituto de Investigación Sanitaria INCLIVA; Blasco-Ibáñez 17, Valencia, Spain; University of Bristol, UNITED KINGDOM

## Abstract

Recent discoveries highlight the importance of stochastic epigenetic changes, as indexed by epigenetic outlier DNA methylation signatures, as a valuable tool to understand aberrant cell function and subsequent human pathology. There is evidence of such changes in different complex disorders as diverse as cancer, obesity and, to a lesser extent, depression. The current study was aimed at identifying outlying DNA methylation signatures of depressive psychopathology. Here, genome-wide DNA methylation levels were measured (by means of Illumina Infinium HumanMethylation450 Beadchip) in peripheral blood of thirty-four monozygotic twins informative for depressive psychopathology (lifetime DSM-IV diagnoses). This dataset was explored to identify outlying epigenetic signatures of depression, operationalized as extreme hyper- or hypo-methylation in affected co-twins from discordant pairs that is not observed across the rest of the study sample. After adjusting for blood cell count, there were thirteen CpG sites across which depressed co-twins from the discordant pairs exhibited outlying DNA methylation signatures. None of them exhibited a methylation outlier profile in the concordant and healthy pairs, and some of these loci spanned genes previously associated with neuropsychiatric phenotypes, such as *GHSR* and *KCNQ1*. This exploratory study provides preliminary proof-of-concept validation that epigenetic outlier profiles derived from genome-wide DNA methylation data may be related to depression risk.

## Introduction

Recent discoveries, mainly in the field of cancer research, highlight the importance of differentially variable methylation signatures as a valuable tool to understand cellular biology [1,2]. Accordingly, new studies are providing biologically-plausible frameworks to understand the origins and implications of such stochasticity-related epigenetic modifications [3–5]. Beyond environmental altering DNA methylation, recent data support the idea of epigenetic stochasticity as an important modifier of DNA methylation. Epigenetic stochasticity refers to the *mutation* of epigenetic marks in the absence of detectable environmental influences, such as in events where DNA methylation marks are not replicated [6,7].

Epigenetic stochasticity, as indexed by DNA methylation variability, has become a very popular candidate mechanism in studies of cancer cell biology [8–10]. Notably, the importance of DNA methylation variability to unravel disease aetiology and dynamics does not seem limited to the field of cancer. For instance, there is some evidence of increased DNA methylation variability in obesity [11] and in depression [12–16]. This clinical background confers particular importance to the study of stochastic epigenetic changes in diseased populations, as they may be linked to aberrant cell function and subsequent human pathology.

Due to the novelty of this subject, only a few tools to statistically assess differences in DNA methylation variability between health and disease have been developed to date [17–19]. Most of these tools have mainly been used in cancer, and incorporate statistical methods in which outlier observations in the healthy and affected DNA samples are purposefully removed before the analyses [17,18]. However, further research has indicated that epigenetic outliers may be frequent across a broad set of pathological states, and could probably function as disease markers [19].

The translatability of the above mentioned techniques to phenotypes apart from cancer comes from previous evidence of a sound epigenetic influence in a wide range of complex phenotypes [7,20]. In this sense, perhaps one of the clearest applications of these approaches outside the field of cancer is the work of Xu et al. [11], who showed an increase in the number of DNA methylation sites with outlier methylation within a group of obese individuals. Nevertheless, applying these methods to pathological states such as mental disorders is not straightforward, since their epigenetic dynamics and the statistical properties of the data extracted from them may have some particularities [16,21]. As described by Mill and Petronis [21], several environmental factors and gene-environment interactions associated with depression are hard to explain: e.g., the prevalence of depression in women almost doubles the prevalence in men after puberty, and depression has a sharp rise in prevalence in women after puberty. Additionally, epigenetic changes observed in psychiatric disorders are quite subtle, i.e. DNA methylation absolute changes reported in the literature are limited, typically under 5%; nevertheless, such small changes may be sufficient to impact mental health [22,23].

As reported by Oh et al. [16] in a sample of monozygotic twin pairs discordant for major depressive disorder, depressed individuals exhibited a statistically significant higher number of epigenetic outliers, in both gene coding and intergenic regions, when compared to healthy subjects. Although these previous findings indicate that there are epigenetic outliers spanning the whole (epi)genome of depressed individuals [16], further research may allow substantiate these findings with standard methods and to determine the precise genomic loci where DNA methylation outliers could be frequent in psychopathology.

The present work aims to confirm the biological feasibility of epigenetic outlier signatures in depressive disorders. To test for variable (outlying) methylation levels at the CpG level associated with depression, the authors analyzed a DNA methylation dataset from the Illumina Infinium HumanMethylation450 Beadchip, which covers >450,000 CpG sites across the human genome. Data for this pilot evaluation came mainly from a set of six monozygotic (MZ) adult twin pairs (12 individuals) discordant in their liability for depressive psychopathology, and groups of concordant and healthy individuals (4 and 7 MZ pairs, respectively) were used to further validate the findings. Using MZ twin samples to analyze methylation variability in disease status has the advantage of suppressing potential sources of methylation variance due to DNA sequence variation. Namely, the potential bias of single nucleotide polymorphism (SNP)-containing probes [24,25] is controlled.

## Results

**Fig 1:**
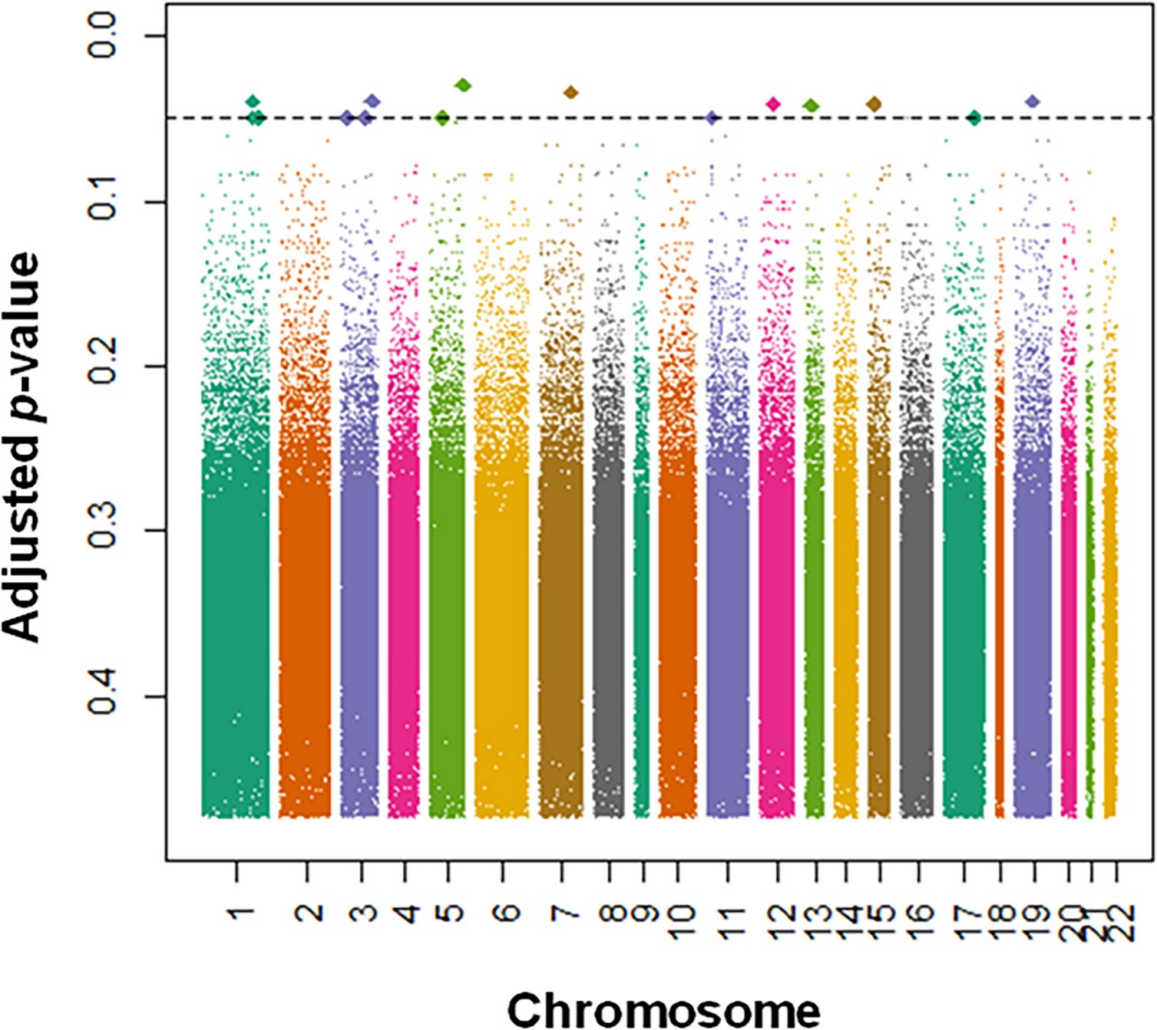
Sixteen DNA methylation probes across the genome exhibit larger methylation variance in the depression-affected co-twins than in their healthy counterparts. The statistical significance of these *p*-values is already adjusted for multiple comparisons using the FDR protocol proposed by Storey and Tibshirani (2003).

After multiple testing adjustments, affected co-twins from discordant MZ pairs showed increased DNA methylation variance at sixteen CpG probes spanning the whole genome (Fig 1).

As described in *Materials and methods*, an additional analysis step was conducted to discard CpG probes showing statistical significance due to potential technical artifacts, or perhaps lacking biological relevance. Specifically, former research indicates that the Illumina technology employed here is able to detect DNA methylation differences of 10% or more with a low probability of error, and there is also evidence showing that methylation differences above 10% are likely to have important functional consequences [13,26–28]. Accordingly, the ranges of DNA methylation values were estimated in both the healthy and affected co-twin subsets, for all 16 CpG probes with increased DNA methylation variance in affected individuals. Since 3 of these probes had only slight increases in DNA methylation ranges (< 10%) in the affected co-twins, they were discarded from the next discussion and analysis steps.

**Fig 2:**
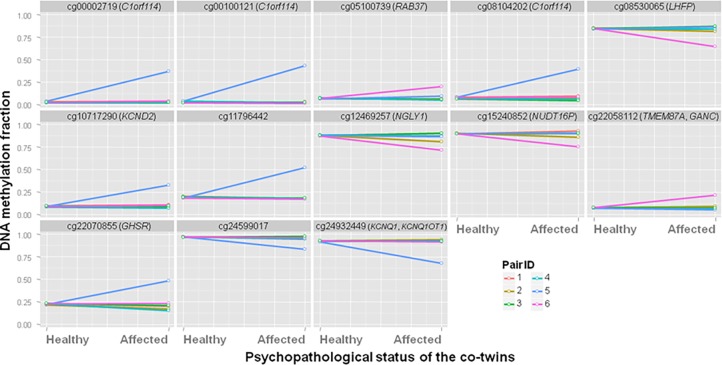
CpG probes with large and statistically significant DNA methylation variances in the diagnostic-discordant MZ twins. The thirteen probes displayed here are those with genome-wide statistically significant methylation variance increases in affected co-twins from the discordant pairs. PairID: randomly assigned pair number.

Fig 2 depicts the DNA methylation values observed in the 6 discordant twin pairs at the remaining 13 probes. Two main observations can be derived from that data. First, the DNA methylation variance increases in depression are driven by epigenetic outliers, rather than by a homogeneous distribution of the methylation values in the affected co-twins (i.e., typically only one of the six affected co-twins constitutes an outlier observation, increasing the overall variance of the group). This fact somehow confirms the feasibility of the adopted statistical protocol (*F*-tests of variance) to detect outlying observations. Secondly, across the 13 probes, it seems that only two out of the six affected co-twins show epigenetic outlier signatures (pairs 5 and 6: blue and pink lines).

Table 1 shows descriptive information on the 13 CpG probes across the genome showing an epigenetic outlier-like profile in depression. The names of the genes they span are also shown, as well as a brief overview of their potential involvement in depression and related brain and behavioral phenotypes.

**Table 1:** DNA methylation probes showing outlier distributions in the affected co-twins from the six adult MZ pairs discordant for depression, and potential neuropsychiatric relevance of their associated genes.

Probe name (TargetID)	Unadjusted *p*-value	Adjusted *p*-value (*q*-value)	*β* range (%): affected	*β* range (%): healthy	*β* range difference	Mean methylation (SD)	Coordinates (hg19)	Gene name (UCSC)	Gene region feature category (UCSC)	Brain-blood methylation correlation*	Potential relevance of the gene in neuropsychiatric disorders
cg00002719	9×10^−7^	0.039	34.8	1.7	33.2	3.9	Chr1:169396706	*CCDC181*	TSS200	None	*CCDC181* methylation has been previously described to be associated with exposure to gestational diabetes mellitus highlighting the importance of prenatal environment in the programming of long-term health and disease [61].
cg00100121	3×10^−6^	0.049	42.1	2.2	39.9	3.6	Chr1:169396635	*CCDC181*	1stExon; 5'UTR	Cerebellum correlation (p = 0.02)	
cg08104202	4×10^−6^	0.049	35.1	2	33	8.4	Chr1:169396712	*CCDC181*	TSS200	None	
cg05100739	4×10^−6^	0.049	15.3	1	14.4	5.7	Chr17:72733163	*RAB37*	TSS200; 1stExon; Body; TSS200	None	Gene expression correlated with brain resting-state oscillatory activity[62].
cg08530065	2×10^−6^	0.042	22.5	1.3	21.3	86.6	Chr13:39980228	*LHFP*	Body	None	Epigenetic regulation of brain function after prenatal insults[63].
cg10717290	3×10^−7^	0.034	25.6	1	24.6	9.2	Chr7:119913576	*KCND2*	TSS200	Prefrontal cortex (p = 0.03) and cerebellum (p = 0.02) correlations	Suggestive evidence of an etiological role in autism[36].
cg11796442	2×10^−6^	0.049	35.2	1.8	33.4	17.8	Chr5:72593919	-	-	None	-
cg12469257	3×10^−6^	0.049	19.2	1.2	18	88.5	Chr3:25761040	*NGLY1*	3'UTR; Body; Body; Body	None	Association with intellectual disability, neuromotor impairment and neuropathy[64].
cg15240852	3×10^−6^	0.049	17.2	1	16.2	90.9	Chr3:131083585	*NUDT16P*	Body	None	-
cg22058112	1×10^−6^	0.041	15.9	1	15.2	7.4	Chr15:42566300	*TMEM87A*; *GANC*	TSS1500; TSS200	None	-
cg22070855	8×10^−7^	0.039	32.9	1.6	31.3	17.4	Chr3:172167527	*GHSR*	TSS1500	Prefrontal cortex (p < 0.001), entorhinal cortex (p = 0.02) and superior temporal gyrus (p < 0.001) correlations	Association with substance abuse[65]. Acts as ghrelin receptor, regulating important features in the central nervous system, such as sleep, mood, memory and reward[31].
cg24599017	1×10^−7^	0.03	14.6	0.4	14.2	96.3	Chr5:178835885	-	-	None	-
cg24932449	4×10^−6^	0.049	26.2	1.6	24.7	91.5	Chr11:2672613	*KCNQ1*; *KCNQ1OT1*	Body; Body	Prefrontal cortex correlation (p = 0.03)	Putative link with working memory, psychopathology and brain activity[33]. DNA methylation levels at birth may correlate with psychiatric symptoms later in life[35].

Note that no SNPs have been described in any of the CpG sites exhibiting outlier distributions. *Brain-blood correlations were retrieved from the *Blood Brain DNA Methylation Comparison Tool*, a publicly available database [56].

**Abbreviations:** TargetID, Illumina identifier; 1stExon, first exon; 5’UTR, 5’ untranslated region; Body, within gene body; TSS200, within 200 bp of a TSS.

**Fig 3:**
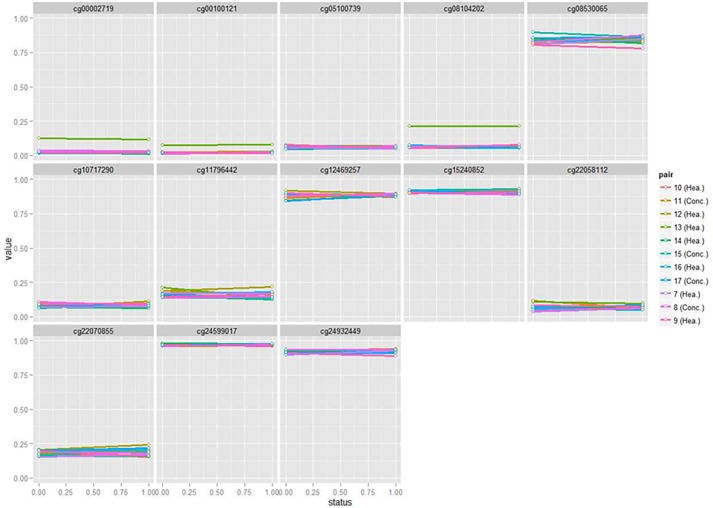
Assessment of DNA methylation levels in diagnostic-concordant and healthy pairs at the 13 CpG probes with epigenetic outlier profiles in affected co-twins from discordant pairs. The thirteen probes displayed here are those with genome-wide statistically significant methylation variance increases in affected co-twins from the discordant pairs (see Fig 2). PairID: randomly assigned pair number.

As an additional validation procedure, the distributions of DNA methylation profiles for the same 13 probes were also analyzed in the subsets of healthy and depression-concordant MZ pairs. If the outlier methylation profiles observed in the affected co-twins from discordant pairs were solely due to technical artifacts or not related to the disease etiology/manifestation, healthy and concordant pairs may show high-variance distributions. The results of these analyses are depicted in Fig 3.

## Discussion

The current study evaluated the feasibility of an epigenetic outlier structure in DNA methylation profiles of depressed individuals. The statistical approach adopted here was customized to account for the fact that, as previously indicated in the literature, both healthy and depressed co-twins may exhibit epigenetic outlier profiles at specific CpG sites across the genome [16]. Most of the CpG sites with outlier distributions in the affected co-twins from depression-discordant pairs were located at genes previously associated with neuropsychiatric and related phenotypes, likely indicating that they have functional consequences on relevant neuropsychiatric pathways. Hence, the results offer a preliminary proof-of-concept validation of a methylation outlier structure in depression, and propose data analysis guidelines to evaluate this epigenetic phenomenon in samples of depressed individuals, where the statistical distribution of DNA methylation levels could differ from other phenotypes.

DNA methylation levels at the thirteen identified CpG probes were retrieved for the groups of diagnostic-concordant and healthy pairs, to explore whether they also exhibited a similar outlier profile regardless of psychopathological status. As shown in Fig 3, most probes exhibit similar ranges of values (and, accordingly, similar variances) across pairs of co-twins, regardless of whether they are concordant or healthy. A number of specificities on the statistical distributions should be noted, as they may provide complementary information. For instance, both co-twins of one of the healthy pairs (pair ID: 13) exhibit an outlier-like profile at CpG sites cg00002719, cg00100121 and cg08104202. However, both co-twins in this pair have almost identical methylation levels at these sites suggesting that their particular genomic DNA sequences may contain low-frequency SNVs associated with hyper-methylation such as SNP-containing probes [24,25]. Alternatively, the shared methylation profile could have arised in response to an environmental exposure common to both co-twins of the pair [29]. Thus, the relatively high methylation levels at these probes are not actually indicating stochastic epigenetic effects in healthy pairs. Rather, the plots would somehow indicate that methylation levels at these three CpG sites can be genetically-regulated, but this observation does not invalidate the epigenetic-outlier pattern observed in the discordant subset. Namely, in the present sample, affected co-twins from discordant pairs showed an outlier profile regardless of their DNA sequence match with their healthy counterparts.

Besides, probes cg08530065, cg11796442, cg12469257 and cg22070855 also show relatively large variances (Fig 3). But as displayed, these CpG sites had similar methylation levels in both co-twins from each pair: the intrapair differences in DNA methylation are typically less than 5%, which may be due to technical measurement artifacts and/or have small functional effects. In this regard, 15 mQTLs have been described to influence cg08530065 methylation, as retrieved from the mQTL database [30]. In contrast, larger methylation differences were observed when comparing the affected co-twin with outlier profile with the healthy co-twin (Table 1 and Fig 2). Hence, analysis of DNA methylation profiles at the thirteen candidate probes (retrieved from the diagnostic-discordant pairs), suggest that outlying methylation profiles are related to diagnostic status. This analysis may also suggest that the outlier profiles are not due to technical artifacts.

Regarding similar research studies, previous population-based clinical reports had used analogous statistical approaches with data from genetically independent individuals with non-psychiatric phenotypes (i.e., singletons; for instance Xu et al. [11]). By definition, DNA sequence variants such as SNPs are equally represented across healthy and depressed co-twin samples. Accordingly, the current study takes advantage of the DNA sequence parity between MZ co-twins discordant for depression to show the presence of epigenetic outliers in affected co-twins, regardless of some SNPs that may be present across the general population.

Additionally, a recent report by Oh et al. [16] has found that epigenetic outliers can be found in both depressed and control populations–though they are more frequent in samples from depressed individuals–. The current report somehow expands on this topic by suggesting that DNA methylation variability due to epigenetic outliers may be related to the neurobiological mechanisms underlying depressive physiopathology. Although there is no clear mechanism about how these epigenetic changes can affect neurobiology downstream, it is important noticing that the identified probes were found within genes and may have relatively direct functional effects on those. Namely, as shown in Table 1, most of the CpG probes found with an epigenetic outlier profile in the affected co-twins from discordant pairs are located within the genomic coordinates of genes previously studied in the literature of psychiatric disorders. In agreement with the findings by Oh et al. [16], our results indicate that DNA methylation variance analyses in depressed individuals should be conducted using one-tailed tests, since some CpG probes with increased variance in normal *control* samples may be present and mislead the biological meaning of the results. Perhaps the most suggestive genes from this set are the ghrelin receptor (*GHSR*), the potassium channel, voltage gated KQT-like subfamily Q, member 1 (*KCNQ1*) and the potassium voltage-gated channel subfamily D member 2 (*KCND2*). Ghrelin plays an important role in a broad spectrum of psychopathological outcomes, including stress, mood-and anxiety disorders [31], probably by modifying brain reward circuitry [32]. Similarly, there is evidence suggesting that *KCNQ1* may be related to psychopathological phenotypes [33], and peripheral tissue DNA methylation levels of *KCNQ1* have been shown to correlate with both adult personality traits [34] and psychiatric symptoms during the first years of life [35]. As opposed to *KCNQ1*, which is predominantly expressed in the adrenal glands and the thyroid, *KCND2* is most expressed in brain tissue; *KCND2* genetic variants have been associated with both epilepsy and autism [36]. Interestingly, CpG probes identified in this set of genes exhibit methylation correlation across blood and brain tissue (see Table 1).

There are several limitations of this study to be noted. First, due to the statistical approach focused in discordant twin pairs, the sample size to estimate DNA methylation outliers was limited to only 6 twin pairs. Each of the epigenetic changes reported here was observed on one discordant pair at a time, suggesting that stochastic factors could be underlying the results, rather than non-shared environment across discordant pairs. However, there is no conclusive evidence against the hypothesis of DNA methylation outliers caused by environmental factors in the current analysis. Additionally, due to the cross-sectional nature of the study and the inclusion of subjects with both prior and current history of anxious-depressive disorders, causality of observed DNA methylation outliers cannot be established. Finally, none of the reported hits have been previously described in association with depression.

In summary, the present results suggest that, alongside other methylation variability mechanisms recently shown in the literature of depression [12–15], epigenetic outliers may index biological disruptions underlying the etiopathology and clinical manifestation of depression.

## Materials and methods

### Subjects

The participants of this study were part of a larger twin sample (UB-Twin Registry) consisting of 242 Caucasian Spanish adult twins from the general population who gave permission to be contacted for research purposes. The exclusion criteria included age under 18 and over 65 years, a medical history of neurological disturbance, presence of sensory or motor alterations and current substance misuse or dependence. Written informed consent was obtained from all participants after a detailed description of the study aims and design, as approved by the Bioethics Committee of the University of Barcelona. All procedures contributing to this work were performed in accordance with the Helsinki Declaration of 1975, as revised in 2008.

Trained psychologists applied face-to-face interviews to apply a battery of psychological and neurocognitive tests and to obtain medical records information. Additionally, peripheral blood or saliva samples were obtained from all 242 participants. The zygosity of the pairs was determined by genotyping 16 highly polymorphic microsatellite loci from DNA samples (SSRs; PowerPlex 16 System Promega Corporation). Identity on all the markers can be used to assign monozygosity with greater than 99% accuracy [37].

A group of 34 middle-aged participants (17 MZ twin pairs; age range 22–56, median age 38; 47% female) who were representative and informative for psychopathology, neurocognition and related factors was extracted from the above-described sample, to be investigated for brain function and genome-wide epigenetic signatures. Peripheral blood was available from all members of this group. Regarding depressive status of the participants, there were 6 discordant, 4 concordant and 7 healthy MZ pairs (12, 8 and 14 individuals). Further information about these participants can be found elsewhere [13,38]; the specific categorical DSM-IV based diagnoses of all subjects are detailed in S1 and S2 Tables. The main analyses described in this manuscript were conducted with the six discordant pairs, and complementary confirmatory analyses were carried out with the concordant and healthy pairs.

### Clinical evaluation

A trained clinical psychologist applied the Structural Clinical Interview for DSM-IV Axis I Disorders (SCID-I) [39] in a face-to-face interview to screen for the presence of any lifetime depression or related anxiety spectrum disorder. Only two out of the twelve participants in this study had predominantly liability for anxious psychopathology. This apparently broad category of disorders was adopted in view of the evidence on comorbidity, shared etiopathology and diagnostic criteria overlap between depressive and anxious disorders [40–44], as well as taking into account evidences of some shared DNA methylation mechanisms in these diagnoses [40,45].

Complementarily, on the day of blood extraction, the current psychopathological status of all participants was evaluated with the Brief Symptom Inventory (BSI) [46,47]. This self-administered 46-item questionnaire is aimed at identifying the experience of psychopathological symptoms during the last 30 days. Its six subscales (depression, phobic anxiety, paranoid ideation, obsession-compulsion, somatization and hostility) were conceived for use in both clinical and non-clinical settings. All items are rated on a 5-point likert scale of distress, according to self-perception of symptoms. There were no between-group differences in intellectual quotient distributions, and the whole sample showed overall intelligence level profiles similar to those reported for demographically analogous samples [48]. Summarized information is shown in Table 2.

**Table 2:** Psychopathological, neurocognitive and demographic variables for DSM-IV diagnostic concordant, discordant and healthy MZ twin pairs.

	CONCORDANT (8 subjects, 8 female)		DISCORDANT (12 subjects, 4 female)		HEALTHY (14 subjects, 4 female)		Group comparison
	Mean (SD)	Range	Mean (SD)	Range	Mean (SD)	Range	X-squared^*a*^; *p*
**Age**	42.5 (13)	22–54	37 (10.9)	20–50	30.3 (7.3)	19–39	5.9; 0.052
**IQ**	105.1 (12.5)	87–127	108.1 (11.8)	87–131	110.5 (5.5)	103–118	1.9; 0.393
**Current psycho-pathology (total BSI)**	27.9 (16.5)	6–57	20.9 (13.3)	4–45	10.6 (9.3)	1–33	8.7; 0.013*
**Current depressive symptoms (BSI subscale)**	6.9 (6.5)	1–20	3.5 (2.7)	0–9	1.7 (1.8)	0–6	6.4; 0.04*

Subjects from discordant twin pairs exhibit intermediate BSI scores (as compared with subjects from healthy or concordant groups) since they constitute a 50% of affected and a 50% of non-affected subjects (their individual scores being averaged).

*Notes*: SD, standard deviation; IQ, intellectual quotient; BSI, Brief Symptom Inventory

^*a*^, Kruskal-Wallis X-squared, as these variables were continuous

*, statistically significant *p*-value.

### Methylation data

The Illumina Infinium HumanMethylation450 (450K) BeadChip [49,50] was employed with peripheral blood DNA samples for all participants. Specifically, by genotyping sodium bisulfite-treated DNA, DNA methylation is assayed by this platform at > 450 000 CpG sites across the genome at single-base resolution; next, bisulfite-converted DNA undergoes whole-genome amplification, before being fragmented and hybridized to microarray probes. The DNA methylation fraction of each CpG site is estimated as *β* = *M* / (*M* + *U* + *α*); *M* and *U* stand for methylated and unmethylated fluorescence intensities, and *α* is an arbitrary offset applied to stabilize *β* values with low intensities.

Infinium methylation data was processed with Methylation Module of GenomeStudio software using HumanMethylation450 manifest v1.1 following the instructions published by Bibikova et al. [49] CpG sites with poor detection quality (*p* > 10^−4^) were removed from further analysis.

The obtained DNA methylation data was further processed to adjust for cell mixture distribution. Briefly, the proportions of different mononuclear cell populations and granulocytes were calculated following a previously published protocol [51,52]. By using 493 probes that matched the informative CpG sites reported by Houseman et al. [51], the proportions of six different cell types (B, CD4^+^ T and CD8^+^ T lymphocytes, plus monocytes, natural killer cells and granulocyte contamination) were estimated across the >450,000 measurements from the Illumina array. Afterwards, a penalized regression procedure allowed retrieving a *β* value representing the *average* cell. A software function to perform this cell mixture adjustment protocol is publicly available at https://gist.github.com/brentp/5058805#file-houseman-r. As expected, results using the adjusted *β* were more conservative than those using the unadjusted methylation values (i.e., there were less statistically significant CpG probes when using the adjusted *β* value).

Since the present MZ twin sample contains both male and female participants, probes in the X and Y chromosomes were removed from the analyses to avoid confounding. Likewise, in view of the relatively small sample size, all CpG probes for which at least one of the 12 diagnostic-discordant individuals had a missing value were removed, giving a final number of 473,864 probes.

The dataset supporting the results of this article have been deposited in NCBI’s Gene Expression Omnibus and is accessible through GEO SuperSeries accession number GSE120307.

### Statistical analyses

In order to find CpG probes in which depressed co-twins from discordant MZ twin pairs exhibited outlier DNA methylation signatures, independent *F*-tests were conducted at each of the 473,864 probes across chromosomes 1 to 22 using var.test() in R. The F-test was implemented using standard procedures as follows. First, let $\bar{X}=\frac{1}{n}\sum_{i=1}^{n}X_{i}$ and $\bar{Y}=\frac{1}{m}\sum_{i=1}^{m}Y_{i}$ be the sample means and $S_{X}^{2}=\frac{1}{n-1}\sum_{i=1}^{n}{\left(X_{i}-\bar{X}\right)}^{2}$ and $S_{Y}^{2}=\frac{1}{m-1}\sum_{i=1}^{m}{\left(Y_{i}-\bar{Y}\right)}^{2}$ be the sample variances. The test statistic is computed as $F=\frac{S_{X}^{2}}{S_{Y}^{2}}$, and it has an F-distribution under the null hypothesis with n-1 and m-1 degrees of freedom. These tests allowed assessing the null hypothesis that the variances of both healthy and affected co-twin groups were equal. This test was chosen to detect epigenetic outlier measurements since it is highly sensitive to departures from normality in a statistical distribution (i.e., outliers) [53]. Considering the evidence of a large number of CpG probes with increased epigenetic outlier features in normal populations when compared to depressed individuals [16], it is necessary controlling for the fact that, in some cases, the control group may display greater variance than the affected group. Hence, one-tailed versions of the *F*-test were implemented.

Multiple testing adjustments were conducted using *q*-values, a measure based upon the false discovery rate (FDR) that has been shown useful in genome-wide statistical analyses and other large-scale multiple comparison settings [54,55]. Values of *q*–the multiple-comparison-adjusted version of *p*–below a 0.05 threshold were considered statistically significant.

An additional filter was applied to the CpG probes obtained from the former procedure. As previous reports indicate that methylation differences above 10% in Illumina assays may have important biological implications and show a low probability of being technical artifacts [13,26–28], a DNA methylation measurement was considered an “outlier” if, apart from being statistically significant at *q* ≤ 0.05, the between-group (healthy vs. depressed) difference in methylation ranges was above 10%.

Information regarding brain and blood correlation of DNA methylation values at the CpG probes meeting the aforementioned outlier criteria was retrieved from the *Blood Brain DNA Methylation Comparison Tool*, a publicly available database [56].

Finally, the names of the genes containing epigenetic outlier probes only within depressed individuals were retrieved to further evaluate the biological feasibility of the results. All analyses, as well as all data visualization procedures, were conducted using some packages for the R software [57–60].

## Supporting information

S1 Table DSM-IV based categorical diagnosis of affected subjects within discordant twin pairs. Pairs 5 and 6, exhibiting outlier methylation profiles have been highlighted in light grey. When a subject met criteria for several categorical entities, those were separated by “/”. Abbreviations: NOS, not otherwise specified.
(DOCX)

S2 Table DSM-IV based categorical diagnosis of subjects within concordant twin pairs. When a subject met criteria for several categorical entities, those were separated by “/”. Abbreviations: NOS, not otherwise specified.
(DOCX)
