# Supplementary material for: Epigenetic outlier profiles in depression: A genome-wide DNA methylation analysis of monozygotic twins
Source: PLoS One. 2018 Nov 20;13(11):e0207754. doi: 10.1371/journal.pone.0207754 (PMC6245788; doi:10.1371/journal.pone.0207754)
Supplement: S1 Table — Pairs 5 and 6, exhibiting outlier methylation profiles have been highlighted in light grey. When a subject met criteria for several categorical entities, those were separated by “/”. Abbreviations: NOS, not otherwise specified. (DOCX) [file pone.0207754.s001.docx]

**S1 Table.** DSM-IV based categorical diagnosis of affected subjects within discordant twin pairs.

| **Pair** | **DSM-IV categorical diagnosis (affected subject)** | **Current vs Lifetime** |
| --- | --- | --- |
| **1** | Depressive Disorder NOS / Agoraphobia without history of panic disorder | Both lifetime |
| **2** | Major Depressive Disorder, Single Episode | Lifetime |
| **3** | Anxiety Disorder NOS | Lifetime |
| **4** | Major Depressive Disorder, Single Episode / Panic Disorder with Agoraphobia | Current / Lifetime |
| **5** | Major Depressive Disorder, Single Episode | Lifetime |
| **6** | Panic Disorder without Agoraphobia | Lifetime |

Pairs 5 and 6, exhibiting outlier methylation profiles have been highlighted in light grey. When a subject met criteria for several categorical entities, those were separated by “/”. Abbreviations: NOS, not otherwise specified.
