# Supplementary material for: Epigenetic outlier profiles in depression: A genome-wide DNA methylation analysis of monozygotic twins
Source: PLoS One. 2018 Nov 20;13(11):e0207754. doi: 10.1371/journal.pone.0207754 (PMC6245788; doi:10.1371/journal.pone.0207754)
Supplement: S2 Table — When a subject met criteria for several categorical entities, those were separated by “/”. Abbreviations: NOS, not otherwise specified. (DOCX) [file pone.0207754.s002.docx]

**S2 Table.** DSM-IV based categorical diagnosis of subjects within concordant twin pairs.

| **Pair** | | **DSM-IV categorical diagnosis** | **Current vs Lifetime** |
| --- | --- | --- | --- |
| Pair 8 | Twin A | Specific phobia / panic disorder without agoraphobia | Both current |
|  | Twin B | Specific phobia / panic disorder with agoraphobia | Current / Lifetime |
| Pair 11 | Twin A | Depressive Disorder NOS | Current |
|  | Twin B | OCD / panic disorder with agoraphobia | Current / Lifetime |
| Pair 15 | Twin A | Panic disorder with agoraphobia / Major Depressive Disorder, Single Episode | Both lifetime |
|  | Twin B | Panic disorder with agoraphobia | Lifetime |
| Pair 17 | Twin A | Major Depressive Disorder, Single Episode / Hypochondriasis | Both lifetime |
|  | Twin B | Major Depressive Disorder, Single Episode / Panic Disorder without agoraphobia | Both lifetime |

When a subject met criteria for several categorical entities, those were separated by “/”. Abbreviations: NOS, not otherwise specified.
